# Research progress on the treatment of knee osteoarthritis combined with osteoporosis by single-herb Chinese medicine and compound

**DOI:** 10.3389/fmed.2023.1254086

**Published:** 2023-09-28

**Authors:** Guanghui Zhou, Xianquan Zhang, Zhuoxu Gu, Jinlong Zhao, Minghui Luo, Jun Liu

**Affiliations:** ^1^The Second Clinical College of Guangzhou University of Chinese Medicine, Guangzhou, China; ^2^The Second Affiliated Hospital of Guangzhou University of Chinese Medicine, Guangzhou, China; ^3^Bone and Joint Research Team of Degeneration and Injury, Guangdong Provincial Academy of Chinese Medical Sciences, Guangzhou, China; ^4^Guangdong Second Traditional Chinese Medicine Hospital, Guangdong Province Engineering Technology Research Institute of Traditional Chinese Medicine, Guangzhou, China

**Keywords:** geriatric medicine, knee osteoarthritis, osteoporosis, bone aging, metabolism, plant-based natural products, signaling pathway

## Abstract

Knee osteoarthritis (KOA) is a degenerative disease with synovial inflammation, articular surface cartilage degeneration, meniscus degeneration, ligament and muscle changes, subchondral bone changes, and osteophyte formation around the joint as the main pathological changes. Osteoporosis (OP) is a disease characterized by low bone mass and deterioration of the microstructure of bone tissue. KOA and OP are both geriatric diseases, and the incidence of KOA combined with OP is high, but there is a lack of specific drugs, and the major treatments are limited to drug therapy. Most traditional Chinese medicine (TCM) treatments use plant-based natural products, and they help patients obtain good clinical benefits and at the same time provide researchers with ideas to study the mechanism of disease occurrence and the relationship between the two diseases. This article summarizes the research progress of TCM monomers and TCM compounds that are frequently used to treat KOA combined with OP to provide ideas for future clinical treatments and related basic research.

## Introduction

Knee osteoarthritis (KOA) and osteoporosis (OP) are common bone and joint degenerative diseases in middle-aged and elderly people. The pathological changes of KOA include synovial inflammation, articular surface cartilage degeneration, meniscal degeneration, ligament and muscle pathologic changes, subchondral bone changes, and periarticular osteophyte formation. OP is a disease characterized by low bone mineral density (BMD) and deterioration of the microstructure of bone tissue. OP results in increasing bone fragility, which increases the risk of fracture. In 1994, the World Health Organization (WHO) established the criteria for measuring BMD, which allows the diagnosis of OP before a fracture occurs. This practical definition is based on its primary and known risk factor, reduced bone strength or density, and includes individuals who are at high risk but do not have fractures (1).

Regarding the relationship between KOA and OP, with the efforts of researchers, the following three viewpoints have widely emerged in recent years: the first viewpoint is that there is a positive correlation between the two diseases, that is, OP can lead to the occurrence of KOA; the second view is that there is a negative correlation between the two diseases, that is, the presence of KOA leads to bone growth, which reduces the occurrence of OP; and the third view is that there is no correlation between the two diseases. However, most studies support a strong correlation between KOA and OP. The two diseases are closely related in epidemiological, pathological, and therapeutic aspects. The majority of patients with KOA are also diagnosed with OP or osteopenia. KOA and OP can interact and eventually form a vicious cycle, which is one of the main causes of knee pain and incapacity for the work of patients (2). A study has shown that the prevalence of KOA combined with OP in China is as high as 30% (2, 3). Therefore, there is a demand for the prevention and treatment of KOA with OP, which we cannot ignore (2).

## Materials and methods

Two researchers conducted a comprehensive search of four databases: China National Knowledge Infrastructure (CNKI), Wanfang Data, China Science and Technology Journal Database (VIP) and PubMed. The search period was up to June 2023 for each database. The four databases were searched by using subject words and free words. The literature retrieval strategy was as follows: “osteoporosis” and “knee osteoarthritis” or “osteoarthritis” and “Chinese medicine.”

## Inclusion and exclusion criteria

The inclusion criteria were as follows: (1) articles mentioned KOA or OP; (2) articles used traditional Chinese medicine (TCM) monomers or TCM compounds for treatment; and (3) there was no restriction on the language of the literature reports.

Exclusion criteria: (1) No reference to TCM treatment; (2) The article only refers to a unique group (e.g., postmenopausal KOA and OP).

## Advantages of TCM in treating KOA and OP

KOA in the understanding of TCM theories belongs to the “Xi Bi” or “Gu Bi” (4, 5), *Huangdi Neijing* recorded: Once external evil invades the human body from the pores or skin, people will have performances of chilling and fear of cold. When the evil invades deep into the bones and remains in the bones, people will have Gu Bi. TCM doctors believe that the occurrence of KOA mostly involves kidney deficiency, external evil, and blood stasis, and kidney deficiency is the primary factor. In TCM theories, OP belongs to the category of “Gu Wei” (5), and TCM practitioners believe that the occurrence of OP also has a certain relationship with the kidney. *Taiping Shenghui Fang* mentioned: The kidney is the residence of shen and jing, and the fullness of yuan qi is closely related to kidney function. If the kidney qi is full, the bones of the human body will be strong. If the qi and blood of the human body is insufficient, viscera dysfunction, the human body’s ability to resist evil, and evil invasion will lead to weakness of kidney qi and then lead to bone marrow that cannot fill the bone, which will lead to Gu Wei and a thin body. Because of the theory that the kidney dominates bone and generates marrow, the treatment of the combination of two diseases in the theories of TCM can start from kidney tonifying, which is a common clinical treatment idea of KOA combined with OP in TCM (6).

TCM has the advantages of flexible means, diverse methods, stable curative effects, etc., in treating KOA and OP. For example, acupuncture, massage, acupotomy, external application of Chinese medicine, and fumigation of Chinese medicine (7, 8). At the same time, TCM drug matching can also take into account other secondary symptoms of patients. If there is a bad appetite in KOA or OP patients caused by spleen and stomach qi stasis, appetizing Chinese medicine such as Radix Aucklandiae, *Citrus reticulata* Blanco or Amomum villosum Lour can be chosen to help patients regulate the spleen and stomach qi and then help restore appetite.

There are many pathogenic factors for the comorbidity of KOA and OP, and the common factors are inflammatory factors, hormonal factors, genetic factors, biomechanical factors, etc. KOA and OP are not immune diseases, but inflammatory factors are involved in bone resorption and cartilage degradation during their course. Related studies have mainly focused on inflammatory factors and hormonal factors. Some researchers have found that nuclear transcription factor-κB (NF-κB) is associated with the pathogenesis and progression of OP and KOA by stimulating the production of various proinflammatory cytokines (such as tumor necrosis factor-α [TNF-α] and interleukin-6 [IL-6]) (9–11). In addition, parathyroid hormone (PTH), estrogen, calcitonin (CT), and other hormones can improve the inflammatory environment of the knee joint, prevent bone mass loss, and delay the progression of the disease (3, 12). At present, nonsurgical treatment is the first choice for the clinical treatment of KOA combined with OP. TCM has obvious advantages in treatment, as it has significant curative effects and fewer side effects than Western medicine treatment, so it has a greater potential promotion value (2). This paper mainly introduces the research progress of TCM monomers and TCM compounds, used frequently by orthopedics and traumatology clinicians, in the treatment of KOA combined with OP and provides directions for future research.

## Treatment of KOA with OP by TCM monomers

The main use method of TCM monomers lies in the effect of tonifying kidney and bone, especially the effect of tonifying kidney Yin or kidney essence. In TCM theories, yang represents the function, and yin represents the substance, so the strength of bone and bone marrow mainly comes from the abundance of yin. This is the reason why TCM doctors should pay attention to the use of bone-tonifying drugs or kidney-tonifying drugs while treating KOA combined with OP. There are many therapeutic active ingredients in TCM monomers, and different effective ingredients play different roles in the treatment of different diseases. The main effective ingredient of TCM monomers mentioned in this paper refers to the known or possible effective active ingredient in the treatment of KOA or OP.

### *Eucommia ulmoides* Oliv

TCM doctors think that *Eucommia ulmoides* Oliv can nourish the liver and kidney, strengthen muscles and bones, and prevent miscarriage. The effective ingredients of *Eucommia ulmoides* Oliv in OP treatment are myrcetin, geniposide, nipinylic acid, etc. Modern pharmacological studies have proven that *Eucommia ulmoides* Oliv can work on the Wnt/β-catenin signaling pathway, bone morphogenetic protein (BMP)/Smad signaling pathway, and mitogen-activated protein kinase (MAPK) signaling pathway. It can promote bone formation and achieve the purpose of treating OP (13). Many scholars have found that the Wnt/β-catenin signaling pathway can influence the proliferation and differentiation of osteoblasts (OBs) and improve their function and activity, which plays a very important role in bone remodeling (14). Relevant studies have shown that the induction of *Eucommia ulmoides* Oliv alcohol extract can promote the osteogenic differentiation of rat bone marrow mesenchymal stem cells and the expression of β-catenin. Relevant animal studies have shown that *Eucommia ulmoides* Oliv can effectively improve the expression of BMP-2 and achieve bone protection in castrated rats (15). Studies have shown that the extract of *Eucommia ulmoides* Oliv, geniposide, and aucubin can activate extracellular signal-regulated kinase (ERK) signaling pathways and then initiate the BMP-2 signaling pathway, inducing the proliferation and differentiation of OB-MC3T3-E1 cells and stimulating bone formation (16). Relevant studies have shown that the main effective ingredients of *Eucommia ulmoides* Oliv in treating KOA include eugenene, eucommia glucoside, eucommia lipid A, quercetin, etc., which are mainly enriched in the MAPK signaling pathway, NF-κB signaling pathway, and Toll-like receptor signaling pathway. KOA can be treated by inhibiting local inflammation and regulating the cell cycle activities of chondrocytes and synovial cells (17).

### *Achyranthes bidentata* Blume

*Achyranthes bidentata* Blume (AB) is commonly used by TCM doctors to treat KOA. TCM doctors believe it can nourish the liver and kidney, strengthen muscles and bones, promote blood circulation, and adjust blood distribution. The effective ingredients of AB Blume in treating OP are quercetin, kaempferol, rhein, etc. Related network pharmacological studies have shown that AB can influence the expression of inflammatory factors such as estrogen receptor gene (ESR), MAPK1, and MAPK14 and delay the progression of OP. ESR1 and ESR2 can participate in the metabolic process of bone cells and effectively inhibit the apoptosis of bone cells. MAPK1 and MAPK14 can promote the proliferation and differentiation of OBs and induce the formation of vascular endothelial cells to prevent and treat OP. Therefore, the prevention and treatment of OP by AB plays a role through multiple targets and pathways (18, 19). Total saponins of Achyranthes are one of the effective ingredients of *Achyranthes bidentata* Blume in the treatment of KOA. Many studies have shown that total saponins of Achyranthes can reduce the levels of interleukin-1 (IL-1) and TNF-α to treat KOA. Meanwhile, total saponins of Achyranthes can influence vascular endothelial growth factor (VEGF) antibodies. VEGF can promote the proliferation of blood vessels in subchondral bone, which can remodel subchondral bone structure and then accelerate the progression of KOA. Related animal experiments have shown that total saponins of Achyranthes could reduce VEGF messenger ribonucleic acid (mRNA) levels in KOA model rabbits, so it can inhibit subchondral bone remodeling (20, 21). The hypoxia inducible factor (HIF) signaling pathway may be the common signaling pathway in KOA and OP.

### Dipsacus asper Wall.ex Henry

Dipsacus asper Wall.ex Henry’s root is a medicine commonly used in orthopedics and traumatology in TCM. Its Chinese name means “continuance of broken bone.” Akebia saponin D (ASD) is the main active ingredient in the treatment of OP (22). Related network pharmacological studies have shown that the main effective active ingredients of Dipsacus asper Wall.ex Henry can be used to treat OP by influencing the occurrence and development of OP through multiple targets and genes. Relevant studies have shown that the target genes of OP treatment by Dipsacus asper Wall.ex Henry mainly involve MAPK14, transforming growth factor-beta 1 (TGF-β1), nitric oxide synthase 2 (NOS2), protein tyrosine phosphatase N1 (PTPN1), coagulation factor II (F2), etc. These genes can influence the localization of proteins in the nucleus, active oxygen metabolism, folding protein reaction, and other biological processes, as well as the activities of functional proteins such as aromatase, carboxylesterase hydrolase, and dopamine transmembrane transporters. Dipsacus asper Wall.ex Henry can postpone the process of OP at the gene level. Studies have confirmed that the activation of ESR1 (ERα) can inhibit the growth of osteoclasts (OCs) and promote the proliferation of OBs, which play a role in the treatment of OP. Similarly, as an important candidate gene for OP susceptibility, TGF-β1 plays a significant role in postponing the occurrence and development of OP by regulating the proliferation and differentiation of bone marrow mesenchymal stem cells and OBs (22, 23). Dipsacus asper Wall.ex Henry still plays a significant role in the treatment of KOA, in which the main active ingredient is Dipsacus total saponins, and relevant animal experiments have pointed out that Dipsacus total saponins may play a role in upregulating the autophagy level of knee chondrocytes in KOA rats by inhibiting the overactivation of the phosphatidylinositol-3 kinase (PI3K)/threonine kinase (AKT)/mammalian target of rapamycin (mTOR) signaling pathway (24). The correlation study on the rate of recombinant factor related apoptosis ligand (Fas-L) expressing positive cells has shown that the rate of synovium cell apoptosis protein B-cell Lymphoma-2 (Bcl-2) and Fas-L expressing positive cells was significantly negatively correlated with treatment of Dipsacus asper Wall.ex Henry. Studies on the correlation between different treatment durations of Dipsacus asper Wall.ex Henry and the rate of cells with positive expression of synovial apoptosis proteins Bcl-2 and Fas-L have shown that the rate of cells with positive expression of synovial apoptosis proteins Bcl-2 and Fas-L is significantly positively correlated with the duration of its treatment, which further indicates that Dipsacus asper Wall.ex Henry has a reliable effect in the treatment of KOA (25).

### Cuscuta chinensis Lam

Cuscuta chinensis Lam in TCM is considered to nourish both the Yin and Yang of the kidney but also tonify kidney essence. The kidney masters bone by nourishing the kidney to achieve the purpose of bone disease treatment. The results of network pharmacology showed that there were 14 components of Cuscuta chinensis Lam for preventing OP, such as quercetin, kaempferol, hypericin, and isorhamnetin. Studies have shown that quercetin can reduce bone loss caused by estrogen deficiency by inhibiting cell senescence. The mechanism of kaempferol may be related to inhibiting bone resorption. Hypericin can promote the proliferation and differentiation of OBs through the BMP and Wnt/β-catenin signaling pathways. The mechanism of isorhamnetin in the prevention and treatment of OP is to regulate the receptor activator of NF-κB ligand (RANKL)/receptor activator of NF-κB (RANK)/osteoprotegerin (OPG) signaling pathway, influence the functions of OBs and OCs, improve the damage to bone microstructure, and prevent and treat OP. It can be seen that the active ingredients of Cuscuta chinensis Lam in preventing OP are effective through different mechanisms of action (26). In terms of KOA treatment, dodder polysaccharide may be the main therapeutic effect of dodder extract on rat KOA in the experiment. The relevant experimental results showed that Cuscuta chinensis Lam extract (including dodder polysaccharide) was selected to act on the rat KOA model, and glucosamine was selected as the positive control group. After 4 weeks, according to behavioral manifestations, changes in joint motion, joint gross morphology, imaging, pathological sections, and other indicators of rats, researchers confirmed that its extract can effectively improve the cartilage degeneration of KOA in rats, and it is more effective than glucosamine. The results indicate that Cuscuta chinensis Lam has a clear therapeutic effect on KOA (27).

## Treatment of KOA with OP by TCM compounds

TCM compounds are combined with TCM monomeric components in accordance with strict compatibility, in which TCM theory has the distinction of Jun, Chen, Zuo, and Shi. While Jun treats the main symptoms of patients, Chen can help patients relieve other symptoms. For example, TCM compounds can help patients treat sleep disorders, such as poor appetite, or other problems while treating bone diseases. TCM compounds embody the integrity principle of TCM and embody the humanistic thought of TCM.

### Zuogui Wan

Modern studies have found that the sensory nervous system regulates bone metabolism at multiple levels. Neuropeptide SP (N-SP) plays an important role in bone cell differentiation, bone metabolism, and bone reconstruction. Both the hypothalamus and bone contain N-SP, and the hypothalamus has the most abundant content, which acts through neurokinin-1 receptor (NK1-R). It has also been found that N-SP can stimulate the proliferation of osteoblast precursor cells, enhance cell activity, and influence bone formation during OB differentiation (28). Runt-related transcription factor 2 (Runx2) is a key gene necessary for bone development that can promote OB and OC differentiation and chondrocyte maturation. Deletion of Runx2 can inhibit OB differentiation and chondrocyte maturation. The expression level of Runx2 in the femur of elderly patients with OP was decreased, and the ability of preosteoblasts to differentiate into OBs was also decreased. The results of studies have shown that compared with the sham operation group, the BMD of the distal femoral subchondral bone of rats in the model group was decreased. Their bone tissue morphology was destroyed, serum inflammatory factors were increased, femur N-SP and Runx2 protein expression was decreased, and hypothalamic neuropeptide N-SP and NK1-R protein and mRNA expression was significantly decreased. This suggests that the morbidity of PMOP combined with OA may be related to the decreased expression of N-SP, NK1-R and Runx2 (29). After intervention with alendronate sodium and Zuogui Wan at different doses, the BMD and bone morphology of rats were improved, the levels of serum inflammatory factors were decreased, the protein expression levels of N-SP and Runx2 in the femur were increased, and the mRNA and protein expression levels of the neuropeptides N-SP and NK1-R in the hypothalamus were increased. The results suggested that sodium alendronate and Zuogui Wan may regulate the expression of N-SP and NK1-R and inhibit the level of serum inflammatory factors, thereby improving the imbalance of bone reconstruction and alleviating cartilage injury. In addition, the Zuogui Wan high-dose group had the best effect on improving BMD and inhibiting serum inflammatory factors. Therefore, the mechanism of Zuogui Wan’s prevention and treatment of PMOP combined with OA may be through upregulating the expression of neuropeptide N-SP and NK1-R in the hypothalamus, thus reversing the imbalance of postmenopausal subchondral bone remodeling. However, the mechanism of its regulation of bone metabolism through the neuropeptide network still needs further study (30).

### Bushen Huoxue decoction

Relevant studies showed that each dose of Bushen Huoxue decoction downregulated the mRNA expression of the inflammatory factors interleukin 1 (IL-1) and IL-6 in the cells of the 3 groups and upregulated the mRNA expression of the cartilage-repairing cytokines insulin-like growth factor 1 (IGF-1) and TGF-β in chondrocytes, with significant differences compared with the normal group. The changes in the expression of these cytokines in the high-dose Bushen Huoxue decoction group were similar to those in the glucosamine hydrochloride and alendronate sodium combined group. Combined with the relevant experimental results, it can be concluded that the downregulated expression of IL-1 and IL-6 and the elevated expression of IGF-1 and TGF-β may play a role in protecting and repairing chondrocytes and promoting chondrocyte proliferation. This verified the positive correlation between the osteoarthritis (OA) and OP groups at the cytotoxic level and verified the therapeutic mechanism of different doses of Bushen Huoxue Decoction on OA and OP and their comorbidities (31, 32). Another study showed that medium and high doses of Bushen Huoxue decoction can downregulate the expression of NF-κB protein in chondrocytes to a certain extent, promote the proliferation of chondrocytes, and reduce the expression of IL-1 and IL-6 in chondrocytes. The effect of the high-dose group was most obvious, thus inhibiting the regulation of NF-κB inflammation to a certain extent. It can reduce the pathological reaction of OA and OP and effectively relieve the clinical symptoms of patients with OA and OP (33).

### Duhuo Jisheng decoction

Duhuo Jisheng Decoction contains Heracleum hemsleyanum Diels, Euzhong, *Achyranthes bidentata* Blume, Angelica sinensis (Oliv.) Diels, Cynanchum otophyllum Schneid, etc. Duhuo Jisheng Decoction can tonify the liver and kidney, promote blood circulation, dispel wind, and remove dampness. Duhuo Jisheng decoction is a common TCM compound used to treat KOA. For arthritis patients, TNF-α is a cytokine produced by activated macrophages that inhibits OBs and stimulates OCs. As a powerful proinflammatory cytokine, TNF-α, an important regulatory cytokine of inflammation and the immune response, can promote the adhesion and migration of inflammatory cells and stimulate the release of IL-1 and adhesion molecules in the body. Basic studies have shown that the levels of IL-1 and TNF-α in the joint fluid of rabbit-diseased joints are decreased after treatment with Duhuo Jisheng Decoction. It can reduce the knee inflammation of patients mainly by affecting IL-1 and TNF-α. Relevant studies have found that the levels of IL-1 and TNF-α at 1, 3, and 5 weeks after treatment with Duhuo Jisheng Decoction are lower than those of patients treated with conventional treatment, and the levels of IL-1 and TNF-α at each time period after treatment with Duhuo Jisheng Decoction show a gradual downwards trend (34, 35).

In addition, Duhuo Jisheng Decoction can be combined with Western medicine to treat KOA with OP. Blood calcium and blood alkaline phosphatase are both bone metabolic indexes that participate in bone formation and are related to the progression of KOA and OP. In the relevant studies, the total effective rate of the study group after treatment with Duhuo Jisheng Decoction combined with Western medicine was higher than that of the control group. The levels of blood calcium and blood alkaline phosphatase were lower than those of the control group, suggesting that Duhuo Jisheng Decoction combined with Western medicine has a good effect on the treatment of KOA combined with OP. It can also improve the bone metabolism of patients. After treatment, the VAS score of the study group was lower than that of the control group, and the quality of life was better than that of the control group, suggesting that Duhuo Jisheng Decoction can effectively relieve joint pain, facilitate the recovery of arthritis and ensure the quality of life of patients. Duhuo Jisheng decoction combined with Western medicine in the treatment of KOA complicated with OP can relieve joint pain in patients and improve bone metabolism indexes and quality of life. Duhuo Jisheng decoction is worthy of clinical promotion and application (36).

## Discussion

In summary, TCM monomers and compounds are effective in treating KOA combined with OP, and the mechanism of TCM in treating OP is realized through systemic, multilink, and multipathway regulation (5). In addition, TCM monomers or compounds can play a therapeutic role in combination with related Western medicines to help expand the scope of treatment. There are various methods for the prevention and treatment of KOA and OP in TCM, which are not limited to the internal administration of TCM monomers and compounds orally but also include Chinese medicine patches, hot compresses, and other means. In addition, acupuncture, massage, traditional exercises, etc., can also be used for preventive health care or treatment (6) or combined rehabilitation training for treatment (37).

However, although the effectiveness of relevant TCM therapies has been confirmed to a certain extent through clinical trials or animal experiments, its main active ingredients and related mechanisms of action are still lacking clear explanations. Meanwhile, the treatment and comprehensive treatment of internal and external TCM treatment lack significant advantages compared with simple Western medicine treatment (6). In addition, there are many separate studies on KOA and OP, but few studies on the combination of the two diseases. Furthermore, relevant TCM studies mainly focus on TCM monomers, TCM compounds, or acupuncture. There is a lack of research on the prevention of traditional exercises, delaying or preventing the progression of KOA combined with OP. Additionally, there are few studies focusing on KOA combined with OP treatment, and most of them mainly focus on the special population of postmenopausal KOA combined with OP. The majority of studies have only focused on patients with KOA or OP patients alone, and KOA combined with OP patients has not received sufficient attention. Furthermore, there are few animal experiments on the relationship between OA and OP (38). Finally, in most trials, the experimental group used Western medicine combined with traditional Chinese medicine, while the control group used Western medicine for comparison, and fewer took placebo or no-treatment control. Although effective decision-making was provided in clinical practice, indicating that the treatment plan of TCM combined with Western medicine was superior to that using Western medicine alone, the efficacy of TCM monomers or compounds alone lacked verification.

In the future, the clinical treatment of KOA or OP should be more balanced because OP is a high-risk factor for the progression of KOA, and KOA can accelerate the progression of OP. More attention can be paid to the treatment from the perspective of regulating bone metabolism (6, 12). In addition, in the clinical treatment of KOA or OP, we should recognize the stage of the disease, stratify patients, make full use of evidence-based medicine, look for higher-level evidence, and consider the stepwise treatment plan (38, 39). In future studies, metabolomics, network pharmacology, and other relevant modern pharmacological research methods can be used to help study the pathogenesis of KOA and OP and clarify the biological characteristics, biological processes, transformation laws, and internal connections of various regulatory mechanisms in the pathological process of KOA combined with OP. They can provide new ideas and a theoretical basis for the prevention and treatment of KOA combined with OP (3). In addition to proving the effectiveness of TCM in the treatment of KOA combined with OP, high-quality clinical experiments and animal experiments should be conducted to study the pathogenesis and pathological changes of KOA and OP. Researchers should expand the study patient group, which can provide a higher level of clinical evidence (38, 40).

In brief, we should make full use of modern pharmacological research to help study the mechanism of action of TCM monomers and TCM compounds in treating KOA with OP and study the effective concentration and side effects on this basis, which can better serve clinical practice. In the clinical practice of Western medicine treatment, clinical practitioners could try to participate in TCM therapy to improve the clinical benefit of patients. The relevant TCM monomers and compounds mentioned in this article are shown in Table 1.

**Table 1:** TCM monomers and compounds used to treat KOA and OP.

	Name	Main active ingredient	Function chennel
TCM monomers	*Eucommia ulmoides* Oliv	OP: myrcetin, geniposide, and nipinylic acid KOA:eugenene, eucommia glucoside, eucommia lipid A, and quercetin, etc.	OP: Wnt/β-catenin signaling pathway, BMP/Smad signaling pathway, and MAPK signaling pathway KOA: MAPK signaling pathway, NF-κB signaling pathway, and Toll-like receptor signaling pathway
	*Achyranthes bidentata* Blume	OP: Quercetin, kaempferol, Rhein, etc. KOA: Total saponins of achyranthes abutens	OP: ESR, MAPK1, MAPK14 KOA: IL-1 and TNF-α
	Dipsacus asper Wall.ex Henry	OP: ASD KOA: Dipsacus Total Saponins	OP: MAPK14, TGF-β1, NOS2, PTPN1, F2, etc. KOA: PI3K/AKT/mTOR signaling pathway, Bcl-2 and Fas-L
	Cuscuta chinensis Lam	OP: quercetin, kaempferol, hypericin and isorhamnetin, etc. KOA: dodder polysaccharide	OP: BMP and Wnt/β-catenin signaling pathway, RANKL/RANK/OPG signaling pathway KOA: unclear
TCM compounds	Zuogui Wan	Unclear	N-SP, NK1-R
	Bushen Huoxue Decoction	Unclear	IL-1, IL-6, IGF-1, TGF-β; NF-κB
	Duhuo Jisheng Decoction	Unclear	IL-1 and TNF-α, etc.

## Conclusion and perspectives

The combination of KOA and OP is common in middle-aged and elderly people, and the participation of traditional Chinese medicine therapy during treatment can help patients improve their quality of life. Compared with simple Western medicine, the participation of Chinese medicine can make the treatment more diversified, making the treatment more individualized and targeted. Future studies should be more inclusive of patient populations and should not be limited to postmenopausal women with KOA and OP. At the same time, more attention is given to animal experiments to provide effective evidence for clinical use. In addition, with the participation of modern pharmacology, finding the common target or pathway of KOA and OP may provide ideas for the development of KOA or OP disease and the study of the relationship between KOA and OP. In clinical practice, more attention should be given to exploring the treatment plan of characteristic TCM therapy for KOA combined with OP so that significant therapeutic effects can be obtained in clinical treatment and patients can obtain higher clinical benefits. In summary, Chinese medicine and Chinese medicine compounds have certain effectiveness in the treatment of KOA combined with OP and can provide ideas for future research on disease development and pathological changes, but more in-depth exploration is needed in specific clinical services.

## Author contributions

GZ: Writing – original draft, Writing – review & editing. XZ: Writing – original draft, Writing – review & editing. ZG: Writing – original draft, Writing – review & editing. JZ: Writing – original draft, Writing – review & editing. ML: Conceptualization, Writing – review & editing. JL: Conceptualization, Writing – review & editing.
